# Outbreak by Hypermucoviscous *Klebsiella pneumoniae* ST11 Isolates with Carbapenem Resistance in a Tertiary Hospital in China

**DOI:** 10.3389/fcimb.2017.00182

**Published:** 2017-05-16

**Authors:** Lingling Zhan, Shanshan Wang, Yinjuan Guo, Ye Jin, Jingjing Duan, Zhihao Hao, Jingnan Lv, Xiuqin Qi, Longhua Hu, Liang Chen, Barry N. Kreiswirth, Rong Zhang, Jingye Pan, Liangxing Wang, Fangyou Yu

**Affiliations:** ^1^Department of Laboratory Medicine, The First Affiliated Hospital of Wenzhou Medical UniversityWenzhou, China; ^2^Department of Laboratory Medicine, The Second Affiliated Hospital of Nanchang UniversityNanchang, China; ^3^Public Health Research Institute Tuberculosis Center, New Jersey Medical School, Rutgers UniversityNewark, NJ, USA; ^4^Department of Laboratory Medicine, The Second Affiliated Hospital of Zhejiang UniversityHangzhou, China; ^5^Department of Intensive Care Unit, The First Affiliated Hospital of Wenzhou Medical UniversityWenzhou, China; ^6^Department of Respiratory Medicine, The First Affiliated Hospital of Wenzhou Medical UniversityWenzhou, China

**Keywords:** *Klesiella pneumoniae*, hypermucoviscous, carbapenem resistance, KPC-2, epidemiology

## Abstract

Hypervirulent and multidrug resistant *Klebsiella pneumoniae* strains pose a significant threat to the public health. In the present study, 21 carbapenem-resistant *K. pneumoniae* isolates (CRKP) were determined by the string test as hypermucoviscous *K. pneumoniae* (HMKP), with the prevalence of 15.0% (21/140) among CRKP, and 1.1% (21/1838) among all *K. pneumoniae* isolates. Among them, 7 (33.3%), and 1 (4.76%) isolate belonged to capsular serotype K20 and K2 respectively, while 13 (61.9%, 13/21) weren't successfully typed by capsular serotyping. All the 21 isolates were carbapenemase-producers and were positive for *bla*_KPC-2_. In addition to *bla*_KPC-2_, all the 21 isolates except one harbor *bla*_SHV-11_, and 15 carry extended-spectrum β-lactamase gene *bla*_CTX-M-65_. The virulence-associated genes with more than 90% of positive rates among 21 isolates included *ureA* (100%, 21/21), *wabG* (100%, 21/21), *fimH* (95.2%, 20/21), *entB* (95.2%, 20/21), *ycf* (95.2%, 20/21), *ybtS* (95.2%, 20/21), and *iutA* (90.5%, 19/21). *rmpA* and *aerobactin* were found in 57.1% (12/21) isolates. Five sequence types (STs) were identified by multilocus sequence typing (MLST), including ST11 (11 K-non capsule typable and 5 K20 isolates), ST268 (1 K20 isolate and 1 K-non capsule typable isolate), ST65 (1 K2 isolate), ST692 (1 K-non capsule typable isolate), and ST595, a novel sequence type (1 K-non capsule typable isolate). Pulsed-field gel electrophoresis (PFGE) results showed two major PFGE clusters, of which cluster A accounts for 6 ST11 isolates (28.6%) and cluster B includes 8 ST11 isolates (38.1%, 8/21). Ten and six ST11 isolates were isolated from 2014 and 2015, respectively, while 8 were isolated from the same month of December in 2014. Ten isolates were collected from the intensive care unit (ICU), and all except one belonged to ST11. Additional 4 ST11 isolates were collected from patients in non-ICU wards, who had more than 10 days of ICU stay history in 2014 prior to transfer to their current wards where the isolates were recovered. Taken together, the present study showed a hospital outbreak and dissemination of ST11 HMKP with carbapenem resistance caused by KPC-2. Effective surveillance and strict infection control strategies should be implemented to prevent outbreak by HMKP with carbapenem resistance in hospitals.

## Introduction

*Klebsiella pneumoniae* is an important human pathogen causing numerous infections in hospitals and long-term care facilities as well as in the communities worldwide, including lung, urinary tract, surgical sites, soft tissues infections and bacteremia (Shon et al., 2013). A new variant of *K. pneumoniae* causing distinctive syndromes such as pyogenic liver abscesses (PLA), designated as hypervirulent (hypermucoviscous) *K. pneumoniae* (HMKP), was initially described from Taiwan in the mid-1980s and 1990s (Liu et al., 1986; Cheng et al., 1991; Wang et al., 1998). HMKP frequently caused severe and life-threatening infections, including endophthalmitis and meningitis in young and healthy individuals, and it is now becoming a global public health problem (Struve et al., 2015). The HMKP strains usually have a distinct hypermucoviscosity phenotype when grown on agar plates (Struve et al., 2015). Since its emergency in Southeast Asia, HMKP has caused sporadic infections in many countries from North America, Europe, South America, Middle East, Australia and Africa (Fang et al., 2005; Siu et al., 2012; Shon et al., 2013; Bialek-Davenet et al., 2014; Yang et al., 2014; Struve et al., 2015; Prokesch et al., 2016). A number of putative virulence genes, including mucoviscosity-associated gene A (*magA*), regulator of mucoid phenotype A (*rmpA*) and aerobactin genes (*aerobactin*), have been found to be associated with HMKP (Fang et al., 2004; Yu et al., 2006; Russo et al., 2014). HMKP is rarely resistant to commonly used antimicrobial agents except for an intrinsic resistance to ampicillin (Lin et al., 2010; Zhang et al., 2016). However, along with the dissemination of mobile genetic elements encoding carbapenemases, carbapenem-resistant HMKP isolates have been increasingly reported (Yang et al., 2014; Yao et al., 2015; Zhang, Y. et al., 2015). The emergence of carbapenem resistant, hypermucoviscous *K. pneumoniae* strains are of great concern as they may be capable of causing severe, untreatable infections in healthy individuals. Indeed, the convergence of enhanced virulence and acquired carbapenem resistance in *K. pneumoniae* strains pose an important threat to the public health. Although, many studies have reported HMKP infections, especially the PLA, the literatures about the prevalence, and molecular characteristics of carbapenem-resistant HMKP isolates are limited. The aim of the present study is to investigate the prevalence of HMKP among CRKP isolates, and to describe the molecular characteristics of carbapenem-resistant HMKP isolates in Wenzhou, eastern China.

## Materials and methods

### Collection and identification of *K. pneumoniae* clinical isolates

From January 2013 to December 2015, a total of 1838 *K. pneumoniae* isolates were consecutively collected from various specimens of patients at the first Affiliated Hospital of Wenzhou Medical University located in Wenzhou, eastern China. These isolates were identified as *K. pneumoniae* by Gram-staining and a VITEK-2 automated microbiology analyzer (bioMérieux, Marcy l'Etoile, France). The control strains, including *Staphylococcus aureus* ATCC25923, *Escherichia coli* ATCC25922, and *Pseudomonas aeruginosa* ATCC27853, were used for control for the species identification. Patient information were extracted from the medical records. This study was approved by the ethical committee of the first Affiliated Hospital of Wenzhou Medical University. Informed consents were obtained from the patients involved in current study.

### Antimicrobial susceptibility testing

Antimicrobial susceptibility testing for agents commonly used for clinical *K. pneumoniae* infection treatment, was examined using a VITEK-2 automated microbiology analyzer platform (bioMérieux, Marcy l'Etoile, France). The minimal inhibitory concentrations (MICs) of imipenem and colistin for imipenem-resistant HMKP were further verified by E-test method according to the guideline recommended by the Clinical and Laboratory Standards Institute (CLSI, 2016). *E. coli* ATCC25922 was used as a control strain for antimicrobial susceptibility testing.

### Detection of ESBLs and KPC enzymes

A modified Hodge test was performed to detect carbapenemases, as described previously (CLSI, 2016). All the isolates were tested for ESBL production by the CLSI-recommended confirmatory double-disc combination test (CLSI, 2016). *Escherichia coli* ATCC 25922 as an indicator bacteria, with sterile saline to *E. coli* ATCC 25922 suspension was adjusted to 0.5 mihms turbidity, and then diluted 10 times with saline. The diluted *E. coli* ATCC 25922 bacterial solution was evenly coated on an MH agar plate and the plate was dried for 3–10 min. Imipenem tablets (10 μg/tablet) was placed in the center of the MH agar plate. Using the 1 μl inoculation ring to pick the test strain or quality control strains to the center of the plate of drug sensitive paper as a starting point, along the centrifugal direction of the line, length 20–25 mm. The plate was incubated at 35°C for 16–20 h.

### Phenotypical identification of HMKP isolates

String test was used to detect hypermucoviscosity phenotype as described previously (Shon et al., 2013). The *K. pneumoniae* isolates with positive hypermucoviscosity phenotypes were designated as HMKP. Briefly, an inoculation loop or needle was used to stretch the bacterial colonies of *K. pneumoniae* isolate on Columbia blood agar plate (BIO-KONT, Wenzhou, China) from overnight culture. The formation of viscous string with >5 mm in length was considered to be positive for the string test.

### Capsular serotyping and detection of resistance genes and virulence-associated genes

The primer sequences for capsular serotyping, and the detection of resistance and virulence genes were listed in Table 1. Capsular serotypes, including K1, K2, K5, K20, K54, and K57, were determined using the methods described previously (Fang et al., 2007; Turton et al., 2010). The presence of resistance genes were detected by PCR amplification followed by Sanger sequencing using the primers and conditions described elsewhere (Pagani et al., 2003; Jiang et al., 2005; Li, H. et al., 2014; Validi et al., 2016). Seventeen virulence-associated genes, including *aerobactin, iroN, kfuB, rmpA, wcaG, alls, ybtS, ureA, uge, wabG, ycf*, *entB, iutA, vatD, magA, fimH*, and *mrkD*, were determined by PCR using the primers described previously for carbapenem-resistant HMKP isolates (Yu et al., 2006, 2008; Turton et al., 2010; Candan and Aksoz, 2015). Bacterial strains harboring these virulence genes from our previous studies were used as positive controls in the PCRs.

**Table 1:** **The sequences of primers for capsular serotyping, resistance genes and virulence-associated genes**.

**Genes**	**Primers**	**Sequence (5′ → 3′)**	**T (°C)**	**Fragment (bp)**	**Refrences**
K1	F	GGTGCTCTTTACATCATTGC	54	1,283	Fang et al., 2007
	R	GCAATGGCCATTTGCGTTAG			Turton et al., 2010
K2	F	GACCCGATATTCATACTTGACAGAG	64	641	Fang et al., 2007
	R	CCTGAAGTAAAATCGTAAATAGATGGC			Turton et al., 2010
K20	F	CGGTGCTACAGTGCATCATT	56	741	Fang et al., 2007
	R	GTTATACGATGCTCAGTCGC			Turton et al., 2010
*bla*_KPC_	F	ATGTCACTGTATCGCCGTCT	55	893	Li, H. et al., 2014
	R	TTTTCAGAGCCTTACTGCCC			Validi et al., 2016
*bla*_SHV_	F	AGCCGCTTGAGCAAATTAAAC	56	713	Jiang et al., 2005
	R	ATCCCGCAGATAAATCACCAC			
*bla*_CTX-M-65_	F	ATGGTGACAAAGAGAGTGCA	54	870	Jiang et al., 2005
	R	CCCTTCGGCGATGATTCTC			Pagani et al., 2003
*ureA*	F	GACAAGCTGTTGCTGTTTACC	58	270	Candan and Aksoz, 2015
	R	CGGGTTGTGAACGGTGAC			
*wabG*	F	ACCATCGGCCATTTGATAGA	58	683	Candan and Aksoz, 2015
	R	CGGACTGGCAGATCCATATC			
*fimH*	F	TGCTGCTGGGCTGGTCGATG	62	909	Candan and Aksoz, 2015
	R	GGGAGGGTGACGGTGACATC			
*entB*	F	ATTTCCTCAACTTCTGGGGC	56	371	Candan and Aksoz, 2015
	R	AGCATCGGTGGCGGTGGTCA			
*ycf*	F	ATCAGCAGTCGGGTCAGC	58	160	Candan and Aksoz, 2015
	R	CTTCTCCAGCATTCAGCG			
*ybtS*	F	CACCGCAAACGCAATCTG	56	782	Candan and Aksoz, 2015
	R	GCCATAGACGCTGTTGTTGA			
*iutA*	F	GGCTGGACATCATGGGAACTGG	66	300	Candan and Aksoz, 2015
	R	CGTCGGGAACGGGTAGAATCG			
*rmpA*	F	ACTGGGCTACCTCTGCTTCA	58	516	Candan and Aksoz, 2015
	R	CTTGCATGAGCCATCTTTCA			Turton et al., 2010
*aerobactin*	F	GCATAGGCGGATACGAACAT	58	556	Yu et al., 2008
	R	CACAGGGCAATTGCTTACCT			
*IroN*	F	GGCTACTGATACTTGACTATTC	58	992	Candan and Aksoz, 2015
	R	CAGGATACAATAGCCCATAG			
*KfuB*	F	GAAGTGACGCTGTTTCTGGC	58	960	Yu et al., 2008
	R	TTTCGTGTGGCCAGTGACTC			
*wcaG*	F	GGTTGGKTCAGCAATCGTA	54	169	Candan and Aksoz, 2015
	R	ACTATTCCGCCAACTTTTGC			Turton et al., 2010
*alls*	F	CCGAAACATTACGCACCTTT	58	508	Candan and Aksoz, 2015
	R	ATCACGAAGAGCCAGGTCAC			
*uge*	F	TCTTCACGCCTTCCTTCACT	60	534	Candan and Aksoz, 2015
	R	GATCATCCGGTCTCCCTGTA			
*vatD*	F	GAAGGAAACAAATCAGTA	48	463	Candan and Aksoz, 2015
	R	GTTTTATTTCGTTAGCAG			
*magA*	F	GGTGCTCTTTACATCATTGC	48	1,149	Candan and Aksoz, 2015
	R	GCAATGGCCATTTGCGTTAG			Yu et al., 2006

### Multilocus sequence typing (MLST)

MLST was performed on all carbapenem-resistant HMKP isolates by amplifying the seven standard housekeeping loci, including *gapA, infB, mdh, pgi, phoE, rpoB*, and *tonB*, as described previously (Diancourt et al., 2005). Sequence types (STs) were assigned using the online database on the Pasteur Institute MLST website (http://bigsdb.pasteur.fr/klebsiella/klebsiella.html).

### Pulsed-field gel electrophoresis (PFGE)

PFGE was performed on all carbapenem-resistant HMKP isolates. In brief, genomic DNA was prepared by embedding *K. pneumoniae* cells in agarose plugs, followed by XbaI digestion for 12 h at 37°C. Electrophoresis was performed at 14°C for 20 h using the Bio-Rad CHEF III system (120° angle, 6V/cm, switch times of 6 and 36 s). *Salmonella* serotype *Braenderup* strain H9812 was used as the molecular marker, and DNA bands were stained with ethidium bromide (0.5 μg/mL). DNA band profiles were interpreted by the criteria of Tenover et al. (1995). Analysis of the PFGE patterns was performed with Bionumerics software (Applied Maths, Sint-Martens-Latem, Belgium) using the Dice Similarity coefficient. The isolates sharing more than 80% similarity were defined as the same PFGE cluster.

## Results

### Prevalence of carbapenem-resistant HMKP isolates

Among 1838 *K. pneumoniae* isolates, 140 (7.6%) were resistant to both imipenem and ertapenem, and were referred as carbapenem-resistant *K. pneumoniae* (CRKP). Among 140 CRKP isolates, 21 (15.0%) were positive for the string test, and were identified as HMKP. 18, 2, and 1 HMKP had imipenem MICs of >32 mg/L, 8 mg/L, and 4 mg/L, respectively. The prevalence of carbapenem-resistant HMKP among all *K. pneumoniae* isolates was 1.1% (21/1838). Interestingly, carbapenem-resistant HMKP was not found in any of the 23 CRKP isolates in 2013. By contrast, 27.9% (12/43) and (12.2%, 9/74) CRKP isolates in 2014 and 2015 were HMKP, respectively. Twenty-one carbapenem-resistant HMKP isolates were recovered from sputum (14 isolates), pus (2 isolates), urine (2 isolates), blood (2 isolates), and drainage (1 isolate), respectively.

### Antimicrobial susceptibility and resistance mechanism

The antimicrobial resistance rates of the 21 carbapenem-resistant HMKP isolates were shown in Table 2. They were all resistant to ampicillin, ampicillin/sulbactam, aztreonam, cefazolin, piperacillin/tazobactam and ceftriaxone. Nineteen (90.5%) of 21 isolates were resistant to ceftazidime and cefotetan, and 80.9% (17/21) were resistant to ciprofloxacin, levofloxacin, gentamicin, tobramycin and cefepime. Sixteen (76.2%, 16/21) and fifteen (71.4%, 15/21) isolates were resistant to amikacin and fosfomycin, respectively. Only one isolate was resistant to sulfamethoxazole/trimethoprim and they were all susceptible to colistin.

**Table 2:** **The antimicrobial resistance profiling of 21 carbapenem-resistant HMKP isolates**.

**Antimicrobials**	**HMKP (*****n*** = **21)**	
	**No**.	**%**
Amikacin	16	76.2
Ampicillin	21	100.0
Amicillin/sulbatam	21	100.0
Aztreonam	21	100.0
Cefazolin	21	100.0
Cefotetan	19	90.5
Ceftriaxone	21	100.0
Cefepime	17	80.9
Ceftazidime	19	90.5
Ceftriaxone	21	100.0
Ciprofloxacin	17	80.9
Colistin	0	0
Imipenem	21	100.0
Levofloxacin	17	80.9
Fosfomycin	15	71.4
TZP^a^	21	100.0
Tobramycin	17	80.9
Gentamicin	17	80.9
SXT^b^	1	4.8

a *TZP, Piperacillin/tazobactam*.

b *SXT, trimethoprim/sulfamethoxazole*.

All 21 HMKP isolates with carbapenem resistance were found to be carbapenemase-producers determined by modified Hodge test. In accordance with the phenotypic results, all isolates were positive for *bla*_KPC-2._In addition to *bla*_KPC-2_, all except one isolates harbored *bla*_SHV-11_, and 15 (71.4%) harbored extended-spectrum β-lactamase gene *bla*_CTX-M-65_.

### Clinical characteristics of 21 patients infected with carbapenem-resistant HMKP isolates

Clinical characteristics of 21 patients infected by carbapenem-resistant HMKP were summarized in Table 3. Among them, 9 were female and 12 were male, with age ranging from 22 to 82 years old. They were from 8 wards including intensive care unit (ICU) (10 cases), neurology ICU (2 cases), gastrointestinal surgery ward (2 cases), traumatology ward (2 case) and other four 4 wards (one case each). However, 4 patients from non-ICU wards had 10–40 days ICU stay history before transfer to their current wards. Fourteen isolates were collected from sputum samples of cases who had pneumonia. Hyperglycaemia was found among 9 cases (42.9%, 9/21). Thirteen cases (61.9%, 13/21) had been treated by carbapenems (imipenem, meropenem or panipenem), and 11 cases (52.4%, 11/21) had surgery. Mechanical ventilation was performed on 15 patients (71.4%, 15/21), and various drainage systems were installed in 14 patients (66.7%, 14/21).

**Table 3:** **Clinical characteristics of 21 patients infected with carbapenem-resistant HMKP isolates^a^**.

	**PFGE type**	**Resistance genes**	**Virulence genes**	**Isolation site**	**Underlying conditions**	**Treatment**	**Invasive procedures**	**Outcome**
B23	A	*bla*_KPC-2_, *bla*_SHV-11_, *bla*_CTX-M-65_	*ureA, wabG, fimH, entB, ycf, ybtS, iutA, rmpA, A*	Sputum	Cancer, encephalorrhagia pneumonia	Panipenem	Mechanical ventilation, drainage system, surgery	Survived
B27		*bla*KPC-2, *bla*SHV-11	*ureA, wabG, fimH, entB, ycf, ybtS, rmpA, A*	Sputum	Encephalorrhagia, pneumonia, hyperglycaemia	TZP	Mechanical ventilation, drainage system, surgery	Giving up treatment
B29		*bla*KPC-2, *bla*SHV-11, *bla*CTX-M-65	*ureA, wabG, fimH, entB, ycf, ybtS*	Sputum	encephalorrhagia pneumonia	TZP, meropenem	Mechanical ventilation, drainage system, surgery	Giving up treatment
B41	C	*bla*KPC-2, *bla*SHV-11	*ureA, wabG, fimH, entB, ycf, ybtS, iutA, rmpA, A*	Sputum	Pneumonia, hypertension, hyperglycaemia	meropenem	Mechanical ventilation	Survived
B44	B	*bla*KPC-2, *bla*SHV-11, *bla*CTX-M-65	*ureA, wabG, fimH, entB, ycf, ybtS, iutA, rmpA, A*	Urine	acute peritonitis, colon cancer, Urinal tract infection	TZP	Mechanical ventilation, drainage system, surgery	Giving up treatment
B49	B	*bla*KPC-2, *bla*SHV-11, *bla*CTX-M-65	*ureA, wabG, fimH, entB, ycf, ybtS, iutA*	Sputum	Head injury pneumonia	SXT, meropenem, levofloxacin	Mechanical ventilation, drainage system	Survived
B50	B	*bla*KPC-2, *bla*SHV-11, *bla*CTX-M-65	*ureA, wabG, fimH, entB, ycf, ybtS, iutA*	Sputum	Cirrhosis, pneumonia	imipenem	Mechanical ventilation, drainage system,	Giving up treatment
B51		*bla*KPC-2, *bla*SHV-11, *bla*CTX-M-65	*ureA, wabG, fimH, entB, ycf, ybtS, iutA*	Sputum	Hemorrhagic shock, pneumonia	SCF, minocycline moxifloxacin	Mechanical ventilation, drainage system	Giving up treatment
B52	B	*bla*KPC-2, *bla*SHV-11, *bla*CTX-M-65	*ureA, wabG, fimH, entB, ybtS, iutA, rmpA, A*	Urine	Urinal tract infection hypertension	TZP	drainage system, surgery	Survived
B53	B	*bla*KPC-2, *bla*SHV-11, *bla*CTX-M-65	*ureA, wabG, fimH, entB, ycf, ybtS, iutA*	Sputum	Encephalorrhagia, pneumonia, hyperglycaemia	Tigecycline SXT, TZP	Mechanical ventilation, drainage system, surgery	Giving up treatment
B56	A	*bla*KPC-2, *bla*SHV-11, *bla*CTX-M-65	*ureA, wabG, fimH, entB, ycf, ybtS, iutA*	Sputum	Pneumonia, colon cancer, heart disease, diabetes mellitus	Meropenem, SXT, teicoplanin	Mechanical ventilation, drainage system	Giving up treatment
B72		*bla*KPC-2, *bla*SHV-11	*ureA, wabG, fimH, entB, ycf, ybtS, iutA, rmpA, A, IroNB*	Sputum	Heart disease, pneumonia, diabetes mellitus	imipenem	Mechanical ventilation:	Giving up treatment
B73	B	*bla*KPC-2, *bla*SHV-11, *bla*CTX-M-65	*ureA, wabG, fimH, entB, ycf, ybtS, iutA, rmpA, A*	Sputum	Head injury, pneumonia, hyperglycaemia	Cefuroxime	Mechanical ventilation, drainage system, surgery	Giving up treatment
B75	B	*bla*KPC-2, *bla*SHV-11, *bla*CTX-M-65	*ureA, wabG, fimH, ycf, ybtS, iutA, rmpA, A*	Sputum	Pneumonia, congenital heart disease	Tigecycline	Mechanical ventilation, surgery	Survived
B77	B	*bla*KPC-2, *bla*SHV-11, *bla*CTX-M-65	*ureA, wabG, fimH, entB, ycf, ybtS, iutA, rmpA, A*	Sputum	Pneumonia	Panipenem, Ofloxacin		Survived
B92		*bla*KPC-2	*ureA, wabG, fimH, entB, ycf, ybtS, iutA, rmpA, A, Kfu, wcaG*	Sputum	Multiple injuries, pneumonia	Imipenem, levofloxacin		Survived
B98	A	*bla*KPC-2, *bla*SHV-11, *bla*CTX-M-65	*ureA, wabG, fimH, entB, ycf, ybtS, iutA, rmpA, A*	Blood	Septic shock, Multiple organ failure	−	Mechanical ventilation	Death
B101	A	*bla*KPC-2, *bla*SHV-11, *bla*CTX-M-65	*ureA, wabG, fimH, entB, ycf, ybtS, iutA*	Pus	acute pancreatitis hyperglycaemia	Meropenem, levofloxacin, linezolid	Mechanical ventilation, drainage system	Giving up treatment
B102	A	*bla*KPC-2, *bla*SHV-11, *bla*CTX-M-65	*ureA, wabG, fimH, entB, ycf, ybtS, iutA*	Pus	Chronic kidney disease peritonitis	Linezolid, SXT meropenem	Drainage system	Giving up treatment
B103	A	*bla*KPC-2, *bla*SHV-11	*ureA, wabG, fimH, entB, ycf, ybtS, iutA, rmpA, A*	Drainage	Perforation of sigmoid colon	imipenem	Drainage system, surgery	Survived
B104	C	*bla*KPC-2, *bla*SHV-11	*ureA, wabG, entB, ycf, iutA, rmpA, A*	Blood	Diabetes mellitus, hypertension	Imipenem, levofloxacin		Survived

a *TZP, piperacillin/tazobactam; SXT, sulfamethoxazole /trimethoprim; A, aerobactin*.

### Capsular serotyping for carbapenem-resistant HMKP isolates

Among the 21 carbapenem-resistant HMKP isolates, 7 (33.3%) belonged to capsular serotype K20, and another one belonged to K2 (4.5%, 1/22). The remaining 13 isolates (61.9%) were not successfully typed, and were therefore defined as K-nontypable.

### Prevalence of virulence-associated genes among carbapenem-resistant HMKP isolates

The virulence-associated genes with more than 90% of detection rates among the 21 isolates included *ureA* (100%, 21/21), *wabG* (100%, 21/21), *fimH* (95.2%, 20/21), *entB* (95.2%, 20/21), *ycf* (95.2%, 20/21), *ybtS* (95.2%, 20/21), and *iutA* (90.5%, 19/21).

### Molecular characteristics of carbapenem-resistant HMKP isolates

**Figure 1:**
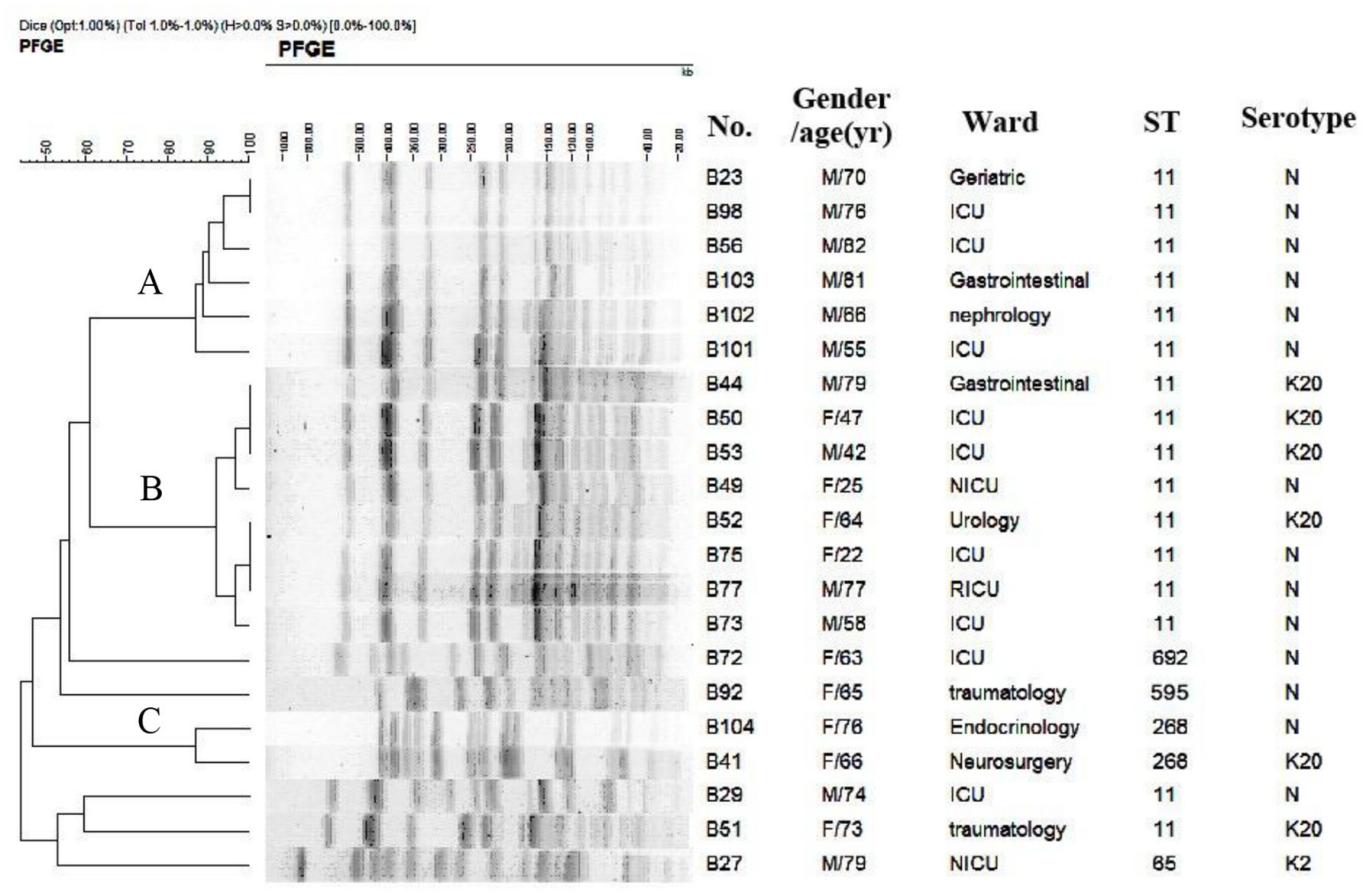
**PFGE results for 21 carbapenem-resistant HMKP isolates**.

Among the 21 carbapenem-resistant HMKP isolates, 5 STs were identified, including ST11 (16 isolates), ST268 (2 isolates), ST65 (1 isolate), ST692 (1 isolate), and ST595, a novel type of STs (1 isolate). PFGE results showed that 16 ST11 isolates were divided into two different PFGE clusters, with cluster A accounting for 6 ST11 isolates (28.6%) and cluster B including 8 ST11 isolates (38.1%, 8/21) (Figure 1). Two ST268 isolates belonged to the same cluster. Each of the remaining 3 isolates formed a singleton.

## Discussion

HMKP has been frequently described in several Chinese hospitals, however, carbapenem-resistant HMKP was rarely found in many previous reports (Li, W. et al., 2014; Liu et al., 2014; Zhang et al., 2016). Here we described the prevalence of HMKP among CRKP in a large tertiary hospital in China, and genetically and phenotypically characterized the carbapenem-resistant HMKP isolates. In this study, the prevalence of HMKP among CRKP isolates is higher than that of 7.4% from two hospitals in Zhejiang Province in China in 2013 (Zhang, R. et al., 2015), but it is similar to the prevalence of 17.9% (5/28) from an another previous report in China (Zhang, Y. et al., 2015). Our study and other reports suggested that acquiring high virulence and carbapenem resistance in *K. pneumoniae* is increasingly becoming a concern in the hospitals in China.

Diabetes mellitus has been considered as a significant risk factor for HMKP infection (Cheng et al., 1991; Wang et al., 1998; Shon et al., 2013; Zhang et al., 2016). In this study, we also identified that 42.9% (9/21) of the patients with HMKP infections had hyperglycaemia. The outcome of patients with infections by carbapenem-resistant HMKP is uncertain. Zhang et al. reported all 5 patients with HMKP infections died of septic shock (Zhang, R. et al., 2015), while another Chinese report showed 4 of 5 patients with similar infections survived (Zhang, Y. et al., 2015). In the present study, 9 patients survived and 1 patient die of septic shock and multiple organ failure, while the remaining 11 patients refused further treatments and requested to be discharged due to the progress of uncontrolled infections or their underlying diseases.

Zhang et al. reported that among 5 carbapenem-resistant HMKP isolates, only one belonged to K2 and the remaining 4 were nontypable (Zhang, R. et al., 2015; Zhang, Y. et al., 2015). Similarly, in this study we only found that one carbapenem-resistant HMKP isolate belonged to K2 (4.5%, 1/22). By contrast, Yao et al. reported that 6 out of 7 carbapenem-resistant HMKP isolates belonged to K2 (Yao et al., 2015). Another Chinese study found that 5 carbapenem-resistant HMKP strains causing fatal infections belonged to K1. K20 hasn't been found to be associated with carbapenem-resistant HMKP isolates before, however, we identified that 33.3% (7/21) of carbapenem-resistant HMKP isolates belonged to K20 in the present study, suggesting that K20 may be an important capsular serotype associated with carbapenem-resistant HMKP.

Although, carbapenem-resistant HMKP isolates were multi-resistant to clinically common-used antimicrobial agents in the present study, sulfamethoxazole/trimethoprim and colistin still had efficient antimicrobial activity *in vitro* against these isolates, indicating that sulfamethoxazole/trimethoprim and colistin could be valuable treatment choices against carbapenem-resistant HMKP infections. *bla*_KPC-2_, which is the main molecular mechanism of carbapenem resistance in *K. pneumoniae* in China, has been previously found to be responsible for carbapenem resistance among HMKP isolates (Zhang, R. et al., 2015). Similarly, all carbapenem-resistant HMKP isolates in the present study were positive for *bla*_KPC-2_.

Previous studies showed that there was a correlation between the presence of *rmpA* gene and virulence in terms of abscess formation of HMKP isolates (Yu et al., 2006; Yang et al., 2014; Yan et al., 2016; Zhang et al., 2016). A Chinese report described that *rmpA* was found in all *K. pneumoniae* isolates causing PLA (Qu et al., 2015). In the present study, 57.1% (12/21) of the carbapenem-resistant HMKP isolates harbored *rmpA*. Meanwhile, previous studies reported that *magA* was associated with the hypermucoviscosity phenotype of *K. pneumoniae*, which was characteristic of the K1 capsular operon (Fang et al., 2004, 2005; Chuang et al., 2006). Yu et al. reported that there was a strong association between *kfuB* and *alls* and K1 isolates, with all K1 isolates positive for *kfuB* and *alls*, and all K2 isolates negative for these two genes (Yu et al., 2008). In this study, all carbapenem-resistant HMKP isolates were negative for *magA, alls*, and *kfuB*, which is consistent with the result that no K1 capsular serotype was identified among the HMKP isolates. *aerobactin* is an important virulence determinant for HMKP, and it has been used as a marker for the identification of HMKP (Zhang et al., 2016). 57.1% (12/21) of the carbapenem-resistant HMKP isolates were found to carry *aerobactin* in the present study, while *iorN* and *wcaG* were only found in one isolate each.

ST11 is the predominant clone of KPC-producing *K. pneumoniae* isolates in China (Qi et al., 2011). However, this epidemic clone was rarely found in HMKP isolates (Liu et al., 2014; Zhang et al., 2016). Zhang et al. reported that ST11 was found in 3 carbapenem-resistant HMKP isolates with unidentified K types (Zhang, Y. et al., 2015). In the present study, ST11 was found to be the most common clone (76.2%, 16/21). Similar to the previous report (Zhang, Y. et al., 2015), 11 out of 16 ST11 isolates were nontypable by the capsular serotyping. However, 5 ST11 isolates belonged to K20, which was firstly reported in current study. Epidemic ST11 strains have been associated with multidrug-resistance and now become hypermucoviscous, which may make the treatment and control for *K. pneumoniae* infections more difficult and poses a major clinical problem for the future. ST23 was the most commonly described ST among HMKP isolates, and was strongly correlated with capsular serotype K1 and liver abscess in several previous studies (Turton et al., 2007; Chung et al., 2008; Shon et al., 2013). A report from China showed that among 5 KPC-2-producing HMKP with K1 serotype, 2 belong to ST23, and 3 belonged to ST1797 (Zhang, R. et al., 2015). In this study, ST23 was not identified, which is consistent with the finding of absence of K1 serotype. Previous studies showed that ST268 was only found among K20 isolates (Liu et al., 2014; Lin et al., 2015; Yan et al., 2015, 2016; Zhang et al., 2016). Similarly, 1 ST268 isolate was found to carry K20 serotype in the present study. In this study, one K2 strain belonged to ST65, which is in accordance with previous study that ST65 was the most common ST associated with K2 serotype in *K. pneumoniae* (Liao et al., 2014). Although, ST692, a two-locus variant of ST65, has been deposited in the *K. pneumoniae* MLST database, it is not reported in published literatures. Our study is the first report of ST692 *K. pneumoniae* with carbapenem resistance associated with clinical infection.

Among 16 ST 11 isolates, the first and second isolates were emerged in January and June, 2014 in our hospital, while 8 (50%) were identified in December, 2014. In 2015, 6 ST11 isolates were identified, and the last isolate was found in October. All 10 except one (ST692) isolates from ICU belonged to ST11. Although, other 4 ST11 isolates were collected from patients in non-ICU wards, they had more than 10 days of ICU stay history in 2014 prior to transfer to their current wards where the isolates were recovered. We suspected that the 4 patients may be initially infected by ST11 carbapenem-resistant HMKP during their stay in ICU, followed by transfer to other wards. The remaining three ST11 isolates were found in neurology ICU (NICU), respiratory ICU (RICU) and nephrology ward. Our data indicated that there was an outbreak of ST11 HMKP with carbapenem resistance in the ICU of our hospital between 2014 and 2015.

In conclusion, the present study reported an outbreak of ST11 HMKP with carbapenem resistance caused by KPC-2 in our hospital. Effective surveillance and strict infection control strategies should be implemented to prevent the dissemination of HMKP with carbapenem resistance, especially outbreak caused by this important pathogen.

## Author contributions

LZ, SW, YG, YJ, JD, ZH, JL, and XQ collected bacteria and performed the experiments. FY, LW, and JP made substantial contributions to conception and design. RZ, LC, and BK revised the manuscript critically for important intellectual content. LH and LC participated experimental design and data analysis. FY drafted the manuscript. All authors read and approved the final manuscript.

## Funding

This work was supported in part by National Institutes of Health (NIH) Grant R01AI090155 (to BK) and R21AI117338 (to LC). The content is solely the responsibility of the authors and does not necessarily represent the official views of the NIH.

### Conflict of interest statement

The authors declare that the research was conducted in the absence of any commercial or financial relationships that could be construed as a potential conflict of interest.
